# Unveiling Antimicrobial and Antioxidant Compositional Differences between Dukkah and Za’atar via SPME-GCMS and HPLC-DAD

**DOI:** 10.3390/molecules27196471

**Published:** 2022-10-01

**Authors:** Mohamed S. Sedeek, Sherif M. Afifi, Mai K. Mansour, Mariam Hassan, Fathy M. Mehaya, Ibrahim A. Naguib, Mohammed A.S. Abourehab, Mohamed A. Farag

**Affiliations:** 1 Pharmacognosy Department, Faculty of Pharmacy, Cairo University, Cairo 11562, Egypt; 2 Pharmacognosy Department, Faculty of Pharmacy, University of Sadat City, Sadat City 32897, Egypt; 3 Department of Medicinal Plants and Natural Products, Egyptian Drug Authority, Giza 11553, Egypt or; 4 Department of Microbiology and Immunology, Faculty of Pharmacy Cairo University, Kasr el Aini st., Cairo 11562, Egypt; 5 Food Technology Department, National Research Center, El-Bohous Str., Dokki, Cairo 12622, Egypt; 6 Department of Pharmaceutical Chemistry, College of pharmacy, Taif University, Taif 21944, Saudi Arabia; 7 Department of Pharmaceutics, Faculty of Pharmacy, Umm Al-Qura University, Makkah 21955, Saudi Arabia; 8 Department of Pharmaceutics, College of Pharmacy, Minia University, Minia 61519, Egypt

**Keywords:** dukkah, za’atar, SPME-GCMS, HPLC-DAD, VP-MIC, DPPH, FRAP, ABTS, ORAC

## Abstract

Interest in plant-based diets has been on the rise in recent years owing to the potential health benefits of their individual components and the notion that plant-based diets might reduce the incidence of several diseases. Egyptian dukkah and Syrian za’atar are two of the most historic and famous Middle Eastern herbal blends used for their anti-inflammatory, hypolipidemic, and antidiabetic effects. Headspace SPME-GCMS and HPLC-DAD were adopted for characterizing the aroma profile and phenolic compounds of both herbal blends, respectively. Further, vapor-phase minimum inhibitory concentration was employed for assessing each blend’s antibacterial potential, while their antioxidant potential was estimated via in vitro antioxidant assays. SPME headspace analysis indicated the abundance of ethers and monoterpene hydrocarbons, while HPLC revealed the presence of several phenolics including rosmarinic acid, ferulic acid, and rutin. Biological investigations affirmed that vapor-phase of the tested blends exhibited antibacterial activities against Gram-positive and Gram-negative pathogens, while the antioxidant potential of the blends was investigated and expressed as Trolox (125.15 ± 5.92 to 337.26 ± 13.84 μM T eq/mg) and EDTA (18.08 ± 1.62 to 51.69 41 ± 5.33 μM EDTA eq/mg) equivalent. The presented study offers the first insight into the chemical profile and biological activities of both dukkah and za’atar.

## 1. Introduction

Chronic illnesses have increased dramatically during the last few decades. Obesity, diabetes, cardiovascular disease, respiratory illnesses, and cancer account for 63 percent of worldwide death each year. Furthermore, chronic illnesses account for almost 45.9% of all disorders globally [1]. People’s health has deteriorated in recent decades, a condition that can be attributed to an unhealthy lifestyle, improper diet, and excessive intake of unrestricted foods and beverages [1]. People consume more than one and a half tons of food per capita during their lifetimes, the composition of which is crucial because nutrition influences 40–60% of diseases [1]. Lifestyle modifications, particularly dietary changes, can be extremely beneficial in preventing, treating, and even reversing a variety of chronic diseases, including coronary artery disease and diabetes [2,3,4]. A plant-based diet consists totally of plant-based foods with a wide range of components. i.e., vegetables, fruits, whole grains, nuts, and seeds [4,5]. Among plant-based diets, Egyptian dukkah and Syrian za’atar are two common examples made entirely of plant ingredients and of potential food (food ingredient, snack, crust; or topping for meals and seasoning for salad) and health benefits (anti-inflammatory, hypolipidemic, and anti-diabetic effects) [6,7].

The Mediterranean diet is regarded as one of the healthiest eating practices. It is considered the most evidence-based diet, where it is capable of preventing and/or treating a variety of ailments. The examination of historic, popular foods, which are widely used as a part of local culture and history, is of particular importance in this context [6]. The Middle Eastern condiments dukkah and za’atar could be regarded as nutritious dietary ingredients owing to the presence of various bioactive components with potentially positive effects on human health.

Because of the growing consumer demand for chemical-free products, researchers have conducted extensive research to assess the feasibility of other preservation techniques, such as the use of essential oils, to improve the microbial quality and safety of food products during their shelf life [8]. Considering their ability to suppress the growth of microorganisms in foods, essential oils obtained from the individual components of dukkah and za’atar (thyme, sumac, coriander, etc.) [9,10,11] have been proposed as food preservatives. Despite their high efficacy against food-borne pathogens, essential oils only have an effect at concentrations exceeding the acceptable flavor thresholds; this may imply an organoleptic impact. Few approaches to lower concentrations have been proposed, such as plant combinations and the use of essential oils in the vapor phase. Essential oils in the vapor phase have potential antibacterial actions against food-borne pathogens, with advantages over essential oils in the liquid phase, such as greater activity at lower concentrations and with no changes in sensory properties of food products [12].

Dukkah, also known as du’ah, do’a, or duqqa, is a traditional Egyptian combination of herbs, nuts, seeds, and spices. Its name is derived from the Arabic word “to pound,” which refers to the ancient practice of pounding spices using a mortar and pestle. It dates back to ancient Egypt but is today extremely popular throughout the Middle East. In Egypt, the term “eeish we dua’ah” is commonly denoted, which literally translates to bread and dukkah, i.e., if someone is experiencing hunger, he or she can always depend on a loaf of bread and some dukkah for dipping [7]. In addition to its nutritional value as a condiment or spice, dukkah has demonstrated a hypoglycemic impact and effectiveness in delaying diabetic complications [7]. While there is no single “traditional” recipe for dukkah, there are some widely accepted foundational ingredients. Seeds (sesame seeds, coriander) and nuts (hazelnuts are the most common frequently combined with almonds, peanuts, and walnuts) are popular garnishes.

Za’atar is another unique flavorful Middle Eastern seasoning blend composed of several blended plants and spices [6]. The Arabic word za’atar describes both a type of herb and a seasoning blend. The za’atar seasoning blend is popular throughout the Middle East, especially in Palestine, Jordan, and Syria. No two recipes are alike, and there are almost certainly many variations following countries’ own cuisines. Nonetheless, three key ingredients define za’atar: sesame seeds, thyme, and sumac [6]. Other herbs and spices found in za’atar include oregano, marjoram, coriander, and cumin. Two varieties of za’atar are available, namely, green za’atar and red za’atar, the primary difference being in the ratio of green herbs (mainly thyme) to red sumac.

Despite the fact that dukkah and za’atar are two of the most famous Middle Eastern herbal blends, and that the beneficial effects of their individual components have been well reported, no studies have defined the chemical profile or bioactivity of the entire herbal mixtures so far in literature. Consequently, this study’s main goal was to characterize the chemical and biological effects of these famous blends, with two varieties of each herbal blend being selected for comparison. Thyme-based Egyptian dukkah (EDT) and nut-based Egyptian dukkah (EDN) were selected as examples for the dukkah mixture, while the za’atar mixture was represented by the green variety, thyme-based Syrian za’atar (SZT), as well as the red variety, sumac-based Syrian za’atar (SZS). Currency inflation and importing restrictions have driven consumers to seek low-cost alternatives; therefore, peanuts have taken the place of more expensive nuts in all commercially available EDN, in contrast to the original recipe. As a result, a common traditional recipe was used to prepare the traditional EDN for examination. The main purpose of this research was to: (1) determine the aroma profile of both dukkah and za’atar; (2) characterize the phenolic profile of the selected blends; (3) screen their antibacterial activity against various resistant bacterial strains; and (4) assess their antioxidant potential via various in vitro assays.

## 2. Results and Discussion

### 2.1. Volatile Components and Their Influence on the Aroma of Egyptian Dukkah and Syrian Za’atar via SPME Analysis

Aroma analysis of Egyptian dukkah and Syrian za’atar was performed to investigate the aroma profile and key aroma compounds of both dukkah and za’atar and determine how the volatile components of the Egyptian dukkah differ from that of Syrian za’atar. Both dukkah’s and za’atar’s aroma compounds consist of several chemical classes; 58 volatiles were identified as belonging to aldehydes, alcohols, carboxylic acids, aromatics, esters, furans, ethers, ketones, nitrogen-containing compounds, oxides, phenols, pyrans/pyrroles, and monoterpene and sesquiterpene hydrocarbons. Quantitatively, the most abundant classes in thyme-based (53.14%) and nut-based dukkah (37.60%) as well as the red variety of za’atar (34.71%) were ethers, confirming their antimicrobial and antioxidant effects, while monoterpene hydrocarbons accounted for the major volatile class in the two za’atar varieties (31.7%). The complete list of identified volatiles in dukkah and za’atar are presented in Table 1.

Alcohols (0.40–1.97%): Five alcohols were detected, with linalool (1.78%) and 4-terpineol (0.73%) being the most abundant, particularly in sumac-based Syrian za’atar and thyme-based Egyptian dukkah, respectively. Linalool, previously detected in fennel [13], and different types of thyme [14] demonstrated significant antibacterial action against *P. fluorescens* [15]. Linalool and terpineol were effective against several tested organisms except *Pseudomonas aeruginosa* [16].

Aldehydes (3.5–10.24%): Aldehydes are commonly identified compounds in the aroma of thyme containing mixtures, and a significant aldehyde increase is observed in most volatiles after the addition of thyme [17]. Aldehydes and their derivatives demonstrated considerable antibacterial activity by mechanically destroying bacterial cell membranes [18]. Cuminaldehyde (0.04–1.94%), one of the major aldehydes in cumin [19], improved antibacterial and anti-biofilm properties in *S. aureus* and *E. coli* when used in conjunction with ciprofloxacin [20]. Anisaldehyde (0.23–7.51%) reported in sumac [21] synergistically improved nisin antimicrobial activity against several resistant food borne pathogens [22].

Esters (0.21–3.3%): Esters are considered to be one of the most important biologically active ingredients in different essential oils compositions [23]. Five esters were found in the four herbal mixes under investigation.

Ethers (16.51–53.14%): Anethole was one of the major identified ethers (7.51–25.40%) that was previously reported as a volatile oil component in thyme [13,24] and was detected as a major constituent in coriander [25]. Anethole-rich volatile oils exerted significant antioxidant and antimicrobial activities [26].

Ketones (1.16–17.38%): Ketones were most abundant in nut-based and thyme-based Egyptian dukkah and were represented by carvone (1.06–14.83%) as the major form. It exceeded 14% of the total volatiles in nut-based Egyptian dukkah. Carvone is the major component of the volatile oil of the family Lamiaceae (including thyme) [27] and was identified as a component of coriander volatile oil [28]. Carvone was proposed to be the most responsible for mentha antibacterial activity against various foodborne pathogenic bacteria [29].

A previous study on 12 fennel accessions demonstrated that anethole and estragole were the main volatiles present at various combinations reaching maximum abundance at 98.4% and 72.4%, respectively. Subsp. piperitum from Minia, Egypt had the highest average estragole exposure at ca. 35 mg/kg body weight for 5 g fennel consumption per day, warranting cautious use in sensitive groups, i.e., nursing mothers and infants [13]. However, our findings indicated much lower estragole level (2.84–35.45%), suggestive of the safe daily use of dukkah and za’atar for prolonged periods. Anethole was also reported as the major volatile in coriander fruits at 85.47% [30]. Moreover, linalool was detected as the main volatile in mature coriander fruits amounting to 87.54% [30]. Our results showed that linalool was detected in sumac-based Syrian za’atar (SZS) at 10-fold higher levels compared to thyme-based Syrian za’atar (SZT), indicative of higher ratios of coriander fruits incorporated in the former herbal blend. Hexanal (56.3%), nonanal (8.7%), and 3-hexen-1-ol (4.4%) were documented as major volatiles in chickpea [31]. Furthermore, hexanal (72.16%) and nonanal (3.24%) constituted the main volatiles in sesame seed [32]. Thus, the content of the aforementioned components in dukkah and za’atar were suggested for a low ratio of chick-pea and sesame incorporated in various herbal blends. Carvacrol (48.5%) and camphor (13.1%) were abundant volatiles previously detected in four thyme accessions [33]. Therefore, SZT encompassed a higher carvacrol content compared to SZS. Likewise, EDT contained more carvacrol compared to EDN. β-Cymene (7.7%), β-ocimene (7.5%), and limonene (7.3%) were reported as chief components in fresh sumac fruit [21], with SZS encompassing higher levels of the aforementioned components compared to SZT.

### 2.2. Phenolic Profile in Egyptian Dukkah and Syrian Za’atar (ug/g)

To complement the profile of volatiles using GC–MS, HPLC was further employed for phenolics profiling in these spice blends to account more for their health benefits and food preservative actions. HPLC revealed that Egyptian dukkah as well as Syrian za’atar were rich in phenolics (Table 2). The thyme-based Egyptian dukkah (EDT) showed the highest levels of identified phenolics such as rosmarinic acid (338.30 ug/g), ferulic acid (325.79 ug/g), and rutin (302.06 ug/g), respectively.

It was obvious that the quantities of these compounds in the various herbal combinations under study varied and accounted for differences in antioxidant activities. Catechin was found at significant levels in EDT and SZT (14.7 and 14.8 ug/g, respectively), but not in EDN. Catechin has very potent antioxidant effects through different mechanisms [34], which could be related to the high antioxidant activity of EDT and SZT using diverse assay techniques.

Caffeic and sinapic acids are two hydroxycinnamates found at substantially higher levels in EDT than in other samples (194.4 and 28.8 ug/g, respectively). They are among the most important hydroxycinnamic acids with strong antioxidant properties [35].

Chlorogenic acid is a polyphenol and an ester of caffeic acid and quinic acid found in the four samples under investigation. Chlorogenic acid and its isomers were shown to have substantial antioxidant and DNA-protective properties [36].

Ferulic acid was found in the four samples with much higher concentrations in EDT, followed by SZT (325.8 and 91.3 ug/g, respectively). Ferulic acid has low toxicity and possesses numerous biological activities (anti-inflammatory, antioxidant, antimicrobial activity, anticancer, and antidiabetic effects) [37].

Rutin, a flavonoid glycoside of quercetin, was detected at 302.1, 12.5, 68.6, and 29 ug/g in EDT, EDN, SZT, and SZS, respectively. Rutin exerts potential antioxidant, anti-inflammatory, and neuroprotective properties, implying a substantial role in the treatment of neurodegenerative and other disease conditions [38].

Apigenin-7-*O*-glucoside is a flavone *O-*glycoside that was detected only in EDT (7.76 ug/g).

Rosmarinic acid, a polyphenol (an ester of caffeic acid and 3-(3,4-dihydroxyphenyl) lactic acid), was found at high levels in both EDT and SZT (338.3 and 149.6 ug/respectively). It has potent antioxidant effects and showed significant cardioprotective activity in diabetic rats [39].

Daidzein and genistin are examples of isoflavonoids, with daidzein found in all samples except SZT, while genistin was detected in all blends except SZS.

### 2.3. Vapor-Phase Minimum Inhibitory Concentration (VP-MIC)

**Figure 1:**
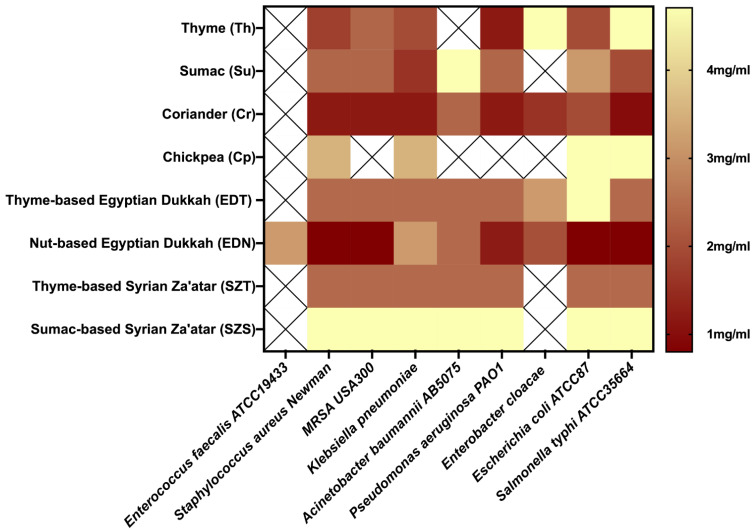
Heatmap showing the vapor-phase antibacterial activity of the tested herbal mixtures and their major individuals. The antibacterial activity is represented by means of vapor-phase minimum inhibitory concentration (VP-MIC). Dark red and yellow represent the lowest and highest VP-MIC (mg/mL), respectively. Symbol “X” means that there was no antibacterial activity detected within the tested concentrations.

Natural antimicrobial compounds have a wide range of applications, particularly in food and biomedical applications [40]. Essential oils’ vapor phase antimicrobial potential has numerous applications in food preservation [41]. As a result, several research studies have been conducted to examine the components and antibacterial activities of essential oils in the vapor phase, owing to the advantages of VP antibacterial assays over direct-contact assays (diffusion or dilution methods) [42]. As an example, EO components are partitioned across the agar in diffusion assays, while in dilution methods, low water solubility must be overcome by the use of emulsifiers or solvents, which may change the action [42]. The vapor-phase of the four mixtures as well as their major components (thyme (Th), sumac (Su), coriander (Cr), chickpeas (Cp)) showed varied antibacterial activity against the highly virulent and resistant tested bacterial strains (Table 3/Figure 1). Interestingly, the vapor-phase of the tested mixtures recorded antibacterial activity against both Gram-positive and Gram-negative pathogens, justifying the use of these herbal blends in food preservation. The tested individuals showed differences in antimicrobial effects against the selected bacterial strains. Coriander was the most potent with VP-MIC ranging from 1.2 ± 0 to 2.4 ± 2 mg/mL (Table 3). Regarding the mixtures, EDN recorded the highest antibacterial activity with the least VP-MIC value against tested pathogens (0.8 ± 0.3 mg/mL) (Table 3; Figure 1). Mixture EDN was the only tested mixture with a vapor-phase that showed antibacterial activity against *Enterococcus faecalis* ATCC19433, with a VP-MIC value of 3.1 ± 1.4 mg/mL. It is worth mentioning that the antimicrobial potential of the EDN exceeded that of individuals against the tested bacterial strains, with the exception of coriander, which showed the lowest VP-MIC (1.2 ± 0 mg/mL) against *Klebsiella pneumoniae* (Table 3). The vapor-phase of mixture SZS recorded the lowest antibacterial activity, with no detected activity against *Enterococcus faecalis* ATCC19433 or *Enterobacter cloacae*. The essential oils of the four herbal blends had effective antibacterial effects in the vapor phase, which was attributed to volatile component dispersion. The presence of carvone [29], furfural, and furfural derivatives [43] could explain EDN’s potential antimicrobial activity. Together with estragole [44] and the other minor constituents, these components could be, at least in part, responsible for the antibacterial activities of EDN. While thyme and coriander have previously been reported to prevent foodborne diseases [45], the synergistic effect between them and the other components of the herbal blends shown in EDN and the other tested mixtures may be a viable option to improve their antimicrobial potential, suggesting the use of Egyptian dukkah and Syrian za’atar in food preservation.

### 2.4. Antioxidant Potential of Egyptian Dukkah and Syrian Za’atar

There are various in vitro chemical models to evaluate the antioxidant potential of the tested mixtures and that are likely to be mediated via phenolic compositions of these herbal blends. The results are shown in Table 4. Radical scavenging capabilities were revealed using DPPH, ABTS, and ORAC and expressed as Trolox equivalent (μM T eq/mg).

The DPPH radical scavenging activities of herbal blend extracts varied from 59.93 to 104.10 μM T eq/mg (Table 4). All tested herbal mixtures exerted scavenging activity, indicating that the components of the herbal mixtures have proton donating ability as well as free radical scavenging ability. STZ showed the highest antioxidant capacity (104.10 μM T eq/mg), followed by SZS (86.79 μM T eq/mg), while EDN showed the lowest antioxidant capacity (26.92 μM T eq/mg). The high concentration of phenolic compounds in SZT (catechin, vanillic acid, rosmarinic acid, and quercetin) may account for its strong DPPH scavenging activity [46].

Regarding the ABTS assay, values varied from 125.15 to 337.26 μM T eq/mg (Table 4). SZT possessed the highest antioxidant capacity (337.26 μM T eq/mg) followed by EDT (225.32 μM T eq/mg). In accordance with the DPPH assay, EDN showed the lowest antioxidant capacity (125.15 μM T eq/mg). Catechin and rosmarinic acids are the major constituents in SZT, detected at 14.8 and 149.6 ug/g, respectively, suggesting that they play a significant role in the antioxidant activity in various assays [47,48].

ORAC values varied from 1138.11 to 1577.86 μM T eq/mg (Table 4). The herbal blend that showed the highest antioxidant capacity was EDT (1577.86 μM T eq/mg) followed by SZT (1479.09 μM T eq/mg). The ORAC assay also confirmed the presence of polyphenols in various blends with significant antioxidant activity [49]. EDT encompassed high levels of caffeic acid, ferulic acid, rutin, and rosmarinic acid (194.4, 325.8, 302.1, and 338.3 ug/g, respectively), demonstrating the strongest antioxidant effect and ROS scavenging activity [48,50]. In this assay, SZS (1138.11 μM T eq/mg) showed the lowest antioxidant potential compared to other tested herbal mixtures.

To confirm the antioxidant potential of herbal blends, ferric reducing antioxidant power (FRAP) was also performed considering its slightly different action mechanism targeting metal chelation and likely mediated via flavonoids found in abundance using HPLC analysis (Table 2). The results did not show much differences from DPPH and ABTS scavenging activities. Similar to the results attained for radical scavenging assays, SZT (212.75 μM T eq/mg) exhibited the highest ferric ion reducing potential versus the lowest, which was exhibited by EDN (59.89 μM T eq/mg).

The ferrozine iron metal chelation power assay was performed to determine the chelation ability of the four herbal blends. Unlike the other assays, the varieties of dukkah exhibited the highest capacities to chelate ferrous ions in comparison to za’atar varieties. EDT (51.69 μM EDTA eq/mg) showed the highest chelation capacity followed by EDN (22.26 μM EDTA eq/mg). The observed iron chelating activity of dukkah’s varieties may be explained by the chelating activity of nut components (hazelnuts, almonds, peanuts, and walnuts) [51]. The chelating activity is attributed to the fact that transition metal ions contribute to oxidative damage in neurodegenerative disorders such as Parkinson’s and Alzheimer’s [52].

It was concluded that Egyptian dukkah and Syrian za’atar are rich in polyphenolics, which accounts for their potential antioxidant and free radical scavenging activities as measured by various assays. The presence of other components in mixtures, as well as differences in the types of major phenolics among blends, resulted in different antioxidant activities. Polyphenolic compounds containing herbs have well-known antioxidant properties [46]. Major phenolics included phenolic acids (caffeic acid, sinapic acid, chlorogenic acid, ferulic acid, and rosmarinic acid), flavonoid aglycones (quercetin and kaempferol), flavonoid glycosides (rutin and apigenin-7-*O*-glucoside), and isoflavonoids (daidzein and genistin). The content of various phenolic components varied across types of Egyptian dukkah and Syrian za’atar, which may be the reason for differences in antioxidant responses depending on the mechanism of compounds present in each sample.

## 3. Material and Methods

### 3.1. Material

#### 3.1.1. Herbal Blends Preparation

Two varieties of each herbal blend were selected for comparison. Except for the nut-based Egyptian dukkah (EDN), all of the tested blends were commercially available. The composition of each blend is listed below:

Thyme-based Egyptian dukkah (EDT): thyme, peanut, chickpea, wheat, and lemon salt.

Nut-based Egyptian dukkah (EDN): chickpea, coriander, sesame, mixture of nuts and apricot seeds, thyme (low percentage), and spices (cumin, salt). An equal proportion of chickpea, coriander, sesame, and a mixture of nuts and apricot seeds was utilized. The nut mixture itself was composed of equal proportions of hazelnuts, almonds, peanuts, walnuts, and apricot seeds. All ingredients were subjected to roasting at 60 °C for 1 h, with stirring before blending.

Thyme-based Syrian za’atar (SZT): sumac, thyme (high percentage), sesame, fennel, anise, coriander, chickpea (also called leblebi or qudamah), and spices (cumin, salt).

Sumac-based Syrian za’atar (SZS): sumac (high percentage), thyme, sesame, fennel, anise, coriander, chickpea (also called leblebi or qudamah), and spices (cumin, salt).

#### 3.1.2. Bacterial Strains

Nine standard strains were tested: *Enterococcus faecalis* ATCC19433, *Staphylococcus aureus* Newman, *Klebsiella pneumoniae* ATCC13883, Methicillin-resistant *Staphylococcus aureus* (MRSA USA300), *Pseudomonas aeruginosa* PAO1, *Acinetobacter baumannii* AB5075, *Escherichia coli* ATCC87, *Enterobacter cloacae*, and *Salmonella typhi* ATCC35664 [53,54,55].

### 3.2. SPME Volatiles Analysis

Headspace volatiles analysis using SPME was adopted from Farag et al. (2021) [56], with a few modifications. Three grams of each sample were placed inside 20 mL clear glass vials. Vials were then immediately capped and placed on a temperature-controlled tray for 30 min at 50 °C with the SPME fiber coated with (DVB/CAR/PDMS, 50/30 μm) 203 divinylbenzene/carboxen/polydimethylsiloxane inserted into the headspace above the sample. A system blank containing no plant material was run as a control.

### 3.3. GCMS Analysis

SPME fiber was desorbed in the injection port of a Shimadzu Model GC-17A gas chromatograph interfaced with a Shimadzu Model QP-5000 mass spectrometer for 1 min at 210 °C (Kyoto, Japan). A DB5-MS column was used to separate volatiles (J&W Scientific, Santa Clara, CA, USA). For 30 s, injections were performed in the splitless mode. The gas chromatograph was used in the manner described by Farag et al. (2021) [56]. At 70 eV, the HP quadrupole mass spectrometer was set to electron ionization mode. The scanning speed was set to 40–500 *m*/*z*. Peaks were deconvoluted using AMDIS software (www.amdis.net; accessed on 12 July 2022) and identified by their retention indices (RI) relative to n-alkanes (C6–C20), mass spectra matching to NIST, the WILEY library database, and authentic standards when possible [57]. For quantification, the relative percentile based on peak area was used as previously reported by Farag et al. (2022) [58].

### 3.4. Determination of Phenolic Compounds Using HPLC-DAD

After placing the sample (0.5 g) in a quick fit conical flask, 20 mL of 2 M NaOH was added, the flasks were flushed with N2, and the stopper was replaced. The samples were stored at room temperature for 4 h. With 6 M HCl, the pH was adjusted to 2. The samples were centrifuged for 10 min at 5000 rpm, and the supernatant was collected. Phenolic compounds were extracted twice with 50 mL of a 1:1 mixture of diethyl ether and ethyl acetate. The organic phase was separated, collected, and evaporated under vacuum at 40 °C using a rotary evaporator before being reconstituted in 2 mL of pure methanol.

A liquid chromatography model 1100 series instrument (Agilent Technologies, CA, USA) equipped with an auto sampler, quaternary pump, and diode-array detector was used for HPLC analysis and phenolics profiling. The phenolic compounds were separated using an Eclipse XDB-C18 analytical column (4.6 × 150 mm; 5 µm) with a Zorbax C18 guard column (4.6 × 12.5 mm; 5 µm). The mobile phase was made up of acetonitrile (solvent A) and 2% acetic acid in water (*v*/*v*) (solvent B). The flow rate was kept at 1 mL/min for a total run time of 65 min, and the gradient program was as follows: 100% B to 87% B in 15 min, 87% B to 85% B in 5 min, 85% B to 78% B in 10 min, 78% B to 60% B in 10 min, 60% B to 40% B in 15 min, 40% B to 0% B in 5 min, and back to initial percent of B in 5 min. The injection volume was 50 µL, and peaks for the benzoic acid and cinnamic acid derivatives, as well as the flavonid, were monitored simultaneously at 280, 320, and 360 nm, respectively. Before injection, all samples were filtered through a 0.45 m syringe filter. Targeted peaks were identified and quantified by matching them with standard materials.

### 3.5. Vapor-Phase Antibacterial Activity of the Tested Mixtures

A group of Gram-negative and Gram-positive highly virulent and multidrug resistant bacterial pathogens was selected to be used as model organisms in testing the vapor-phase antibacterial activity of the studied mixtures. The selected pathogens are members of the "ESKAPE pathogens" that are attracting research attention, being the top leading causes of life-threatening infections [53].

#### Vapor-Phase Minimum Inhibitory Concentration (VP-MIC)

**Figure 2:**
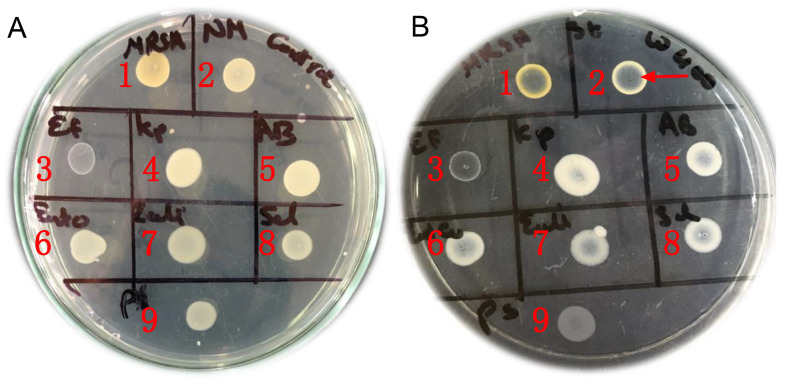
Vapor-phase minimum inhibitory concentration assay (VP-MIC). (**A**) Photo of the control plate showing the bacterial growth of the nine selected microorganisms. (**B**) Photo of the sample plate showing inhibition of bacterial growth. The numbers 1–9 on photos (**A**) and (**B**) correspond to the tested microorganisms as follows: 1. Methicillin-resistant *Staphylococcus aureus* (MRSA USA300), 2. *Staphylococcus aureus* Newman, 3. *Enterococcus faecalis* ATCC19433, 4. *Klebsiella pneumoniae* ATCC13883, 5. *Acinetobacter baumannii* AB5075, 6. *Enterobacter cloacae*, 7. *Escherichia coli* ATCC87, 8. *Salmonella typhi* ATCC35664, and 9. *Pseudomonas aeruginosa* PAO1. The arrow in photo B2 shows growth suppression of the tested microorganism. The VP-MIC is identified as the least concentration of the tested mixture that causes growth suppression of the tested microorganism when compared to the control.

The vapor-phase antibacterial activity of the tested mixtures was investigated by determination of the vapor-phase minimum inhibitory concentration (VP-MIC) adopting the principles and approaches described before [12,59]. The previously established methods for the determination of VP-MIC used either the disc volatilization assay or the airtight box assay [12,59]. The disc volatilization assay was not applicable to our samples since we were dealing with mixtures of powders/solid particles instead of dealing with essential oils (liquid samples). The airtight box assay was thus found more convenient with our samples but with the drawback of consuming a large number of materials (culture media, inoculum, samples, etc.). The previously established method was thus slightly modified to develop a cost-efficient method that could test a large number of microorganisms using the least amount of samples using the conventional petri dish and without the need for any specialized tools. The VP-MIC of the tested mixtures was determined as follows: 15 mL of sterile Mueller–Hinton agar (MHA) was pipetted in a 10 cm diameter glass petri dish. The solidified MHA surface was then surface inoculated by spotting 10 μL of the tested bacterial suspension (106 CFU/mL). Since nine microorganisms were tested in each assay, 10 μL of each bacterial inoculum was spotted side by side in the same petri dish, as shown in Figure 2. The tested mixture was then placed on the cover of the petri dish, and the plate was kept inverted so that the inoculated agar was upward and the cover with the tested mixture was downward. Later, the petri dish was sealed by parafilm and incubated at 37 °C for 24 h. The control plate was prepared in the same way but keeping the cover of the petri dish empty without adding any samples. For each mixture, several plates were prepared with different concentrations (4.7, 2.4, 1.8, 0.6 mg/mL). The concentration of the tested mixture was calculated by dividing the weight (mg) of the mixture (placed on the cover of the petri dish) by the volume (mL) of the airspace in the petri dish. After incubation, the bacterial growth in the sample (mixture) plates and control plate was compared, and the VP-MIC was determined. The VP-MIC was identified as the least concentration of the tested mixture that caused apparent growth suppression of the tested microorganism when compared to the control (Figure 2). The assay was repeated at least three independent times, and the VP-MIC was reported as mean ± SD (standard deviation).

### 3.6. Antioxidant Potential of the Tested Herbal Mixtures

**Figure 3:**
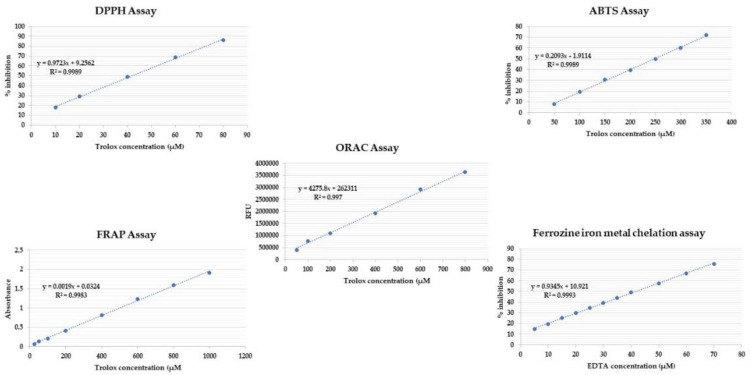
Standard calibration curves of the antioxidant assays expressed as Trolox and EDTA.

Several in vitro antioxidant assays were conducted on the crude methanolic extract of each blend and expressed as Trolox and EDTA. The calibration curve for the standard of each assay is shown in Figure 3.

#### 3.6.1. DPPH Free Radical Scavenging Activity

Scavenging of free radicals such as DPPH is the most employed antioxidant assay to determine the antioxidant capacity of the plant extracts [60]. Except for sample EDN, which was prepared at a concentration of 1 mg/mL, all extracts were prepared in 100% methanol at a concentration of 0.5 mg/mL. In methanol, a stock solution of 100 μM Trolox was obtained, from which 7 concentrations of 80, 60, 40, 20, and 10 μM were prepared (Figure 3). The DPPH (2,2-diphenyl-1-picryl-hydrazyl-hydrate) free radical assay was conducted using the method described by Boly [61]. In brief, 100 μL of freshly prepared DPPH reagent (0.1 percent in methanol) was mixed with 100 μL of sample in a 96-well plate (*n* = 6), and the reaction was incubated at room temperature for 30 min in the dark. The reduction in DPPH color intensity was measured at 540 nm at the end of the incubation time. Data are represented as means ± SD according to the following equation: percentage inhibition = ((average absorbance of blank − average absorbance of the test)/(average absorbance of blank)) × 100. The results were recorded using a FluoStar Omega microplate reader (BMG LABTECH, Berlin, Germany).

#### 3.6.2. ABTS Assay

The ABTS assay, also known as the Trolox equivalent antioxidant capacity (TEAC) assay, compares the ability of antioxidants to scavenge ABTS in the aqueous phase to a Trolox standard [62]. The extracts were prepared at the concentration of 0.5 mg/mL in methanol, except for sample EDN, which was prepared at the concentration of 1 mg/mL. A Trolox stock solution of 1 mM in methanol was prepared, and 5 serial dilutions were prepared in the concentrations of 400, 350, 300, 250, 200, 150, 100, and 50 μM (Figure 3). The assay was carried out according to the method of Arnao [63], with minor modifications carried out in the microplates. Briefly, 192 mg of ABTS was dissolved in distilled water and transferred to a 50 mL volumetric flask, and then the volume was completed with distilled water. Then 1 mL of the previous solution was added to 17 μL of 140 mM potassium persulphate, and the mixture was left in the dark for 24 h. After that, 1 mL of the reaction mixture was completed to 50 mL with methanol to obtain the final ABTS dilution used in the assay. Then 190 μL of the freshly prepared ABTS reagent was mixed with 10 μL of the sample/compound in 96-well plates (*n* = 6), and the reaction was incubated at room temp. for 30 min in dark. At the end of the incubation time, the decrease in ABTS color intensity was measured at 734 nm. Data were represented as means ± SD according to the following equation: percentage inhibition = ((average absorbance of blank − average absorbance of the test)/(average absorbance of blank)) × 100.

#### 3.6.3. Oxygen Radical Absorbance Capacity (ORAC) Assay

ORAC provides a tool for preliminary evaluation and screening of antioxidant potential of herbal extracts [64]. The extracts were prepared at the concentration of 0.4 mg/mL in methanol. A Trolox stock solution of 1 mM in MeOH was prepared, and 6 serial dilutions were prepared in the concentrations of 800, 600, 400,200, 100 and 50 μM (Figure 3). The assay was carried out according to the method of Liang [65], with modifications; briefly, 10 μL of the prepared sample(s) was incubated with 30 μL fluorescein (100 nM) for 10 min at 37 °C. Fluorescence measurement (485 EX, 520 EM, nm) was carried out for three cycles (cycle time, 90 s) for background measurement. Afterward, 70 μL of freshly prepared 2,2′-azobis(2-amidinopropane) dihydrochloride (AAPH) (300 mM) was added immediately to each well. Fluorescence measurement (485 EX, 520 EM, nm) was continued for 60 min (40 cycles, each 90 sec). Data were represented as means (*n* = 3) ± SD, and the antioxidant effect of the compound/extract was calculated as μM Trolox equivalents by substitution in the linear regression equation y = 4275.8x + 262311. The results were recorded using a FluoStar Omega microplate reader (BMG LABTECH, Germany).

#### 3.6.4. The Ferric Reducing Ability of Plasma (FRAP Assay)

The ferric reducing antioxidant power (FRAP) assay is an antioxidant capacity assay that measures antioxidants’ ability to reduce ferric ion (Fe^3+^)–ligand complexes to ferrous (Fe^2+^) complexes in an acidic medium [66] and provide a reliable tool to measure the combined activity of redox-active antioxidants [67]. In methanol, extracts were prepared at a concentration of 5 mg/mL. A Trolox stock solution in methanol was prepared at a concentration of 2 mM, and the following dilutions were prepared: 1000, 800, 600, 400, 200, 100, 50, and 25 M (Figure 3). The assay was carried out using Benzie’s method [68], with minor modifications to be carried out in microplates. A freshly prepared TPTZ reagent (300 mM acetate buffer (pH = 3.6), 10 mM TPTZ in 40 mM HCl, and 20 mMFeCl_3_, respectively, in a ratio of 10:1:1 *v/v/v*) was used. In a 96-well plate (*n* = 3), 190 uL of freshly prepared TPTZ reagent were mixed with 10 uL of sample, and the reaction was incubated at room temperature for 30 min in the dark. At the end of the incubation period, the resulting blue colored sample was measured at 593 nm. The ferric reducing ability of the samples was presented as μM TE/mg sample using the linear regression equation extracted from the linear dose–response curve of Trolox: y = 0.0014x + 0.1639. Data are presented as means ± SD recorded using a FluoStar Omega microplate reader (BMG LABTECH, Germany).

#### 3.6.5. Ferrozine Iron Metal Chelation Assay

The transition metal ion Fe^2+^ has the ability to sustain the formation of free radicals through electron gain or loss. As a result, the chelation of metal ions with chelating agents can reduce the formation of reactive oxygen species [69]. Sample EDT was prepared at the concentration of 0.5 mg/mL in methanol, while the other samples were prepared at 2.5 mg/mL. An EDTA stock solution of 0.1 mM was prepared in water, and 11 serial dilutions were prepared in the concentrations of 5, 10,15, 20, 25, 30, 35, 40, 50, 60, and 70 μM (Figure 3). The assay was carried out according to the method of Santos [70], with minor modifications to be carried out in microplates; briefly, 20 μL of the freshly prepared ferrous sulphate (0.3 mM) were mixed with 50 μL of the sample/compound in 96-well plates (n = 6). Afterwards, 30 μL of ferrozine (0.8 mM) was added to each well. The reaction mixture was incubated at room temperature for 10 min. At the end of incubation time, the decrease in the produced color intensity was measured at 562 nm. Data were represented as means ± SD according to the following equation: (1) percentage inhibition = ((average absorbance of blank − average absorbance of the test)/(average absorbance of blank)) × 100. The results were recorded using a FluoStar Omega microplate reader (BMG LABTECH, Germany).

## 4. Conclusions

The aroma profiles, phenolic compounds, and antibacterial and antioxidant effects of Egyptian dukkah and Syrian za’atar were evaluated in this study. Attempts to characterize essential oil compositions of the herbal blends revealed the abundance of ethers and monoterpene hydrocarbons in dukkah and za’atar, respectively. Alcohols, aldehydes, aromatics, carboxylic acids, esters, furans, ketones, nitrogen-containing compounds, oxides, phenols, pyrans, pyrroles, and sesquiterpene hydrocarbons were also detected. HPLC further revealed that both dukkah and za’atar were rich in phenolics, with rosmarinic and ferulic acids as the most abundant, especially in the thyme-based Egyptian dukkah (EDT). Regarding the vapor-phase of the four herbal blends, blends showed variable antibacterial activity, with nut-based Egyptian dukkah (EDN) recording the highest antibacterial activity against the tested pathogens, and sumac-based Syrian za’atar (SZS) recording the lowest antibacterial activity. The in vitro assays affirmed the antioxidant potential of the four herbal blends. Thyme-based za’atar (SZT) showed the highest DPPH and ABTS scavenging activities as well as the highest ferric ion reducing potential, while thyme-based Egyptian dukkah (EDT) showed the highest ORAC capacity followed by the highest chelation capacity. The presence of thyme may explain za’atar’s enhanced free radical scavenging antioxidant activities, while the presence of nuts may explain the chelation activity as well as the potentiation of dukkah’s antimicrobial activities. Further studies are needed to provide the complete chemical characterization of Egyptian dukkah and Syrian za’atar, as well as to demonstrate the beneficial effects of the two herbal blends as typical examples for plant-based diets. Further research is also required to investigate the herbal blends for other potential biological activities and to examine how much a synergized effect is observed compared to herbal blends of individual components regarding other effects as revealed from antimicrobial assays.

## Figures and Tables

**Table 1:** Relative percentage of volatile components in Egyptian dukkah and Syrian za’atar analyzed using headspace SPME-GCMS. Each presented value is a mean ± SD (*n* = 3).

No.	RT (min)	Name	RI	EDT ^a^	EDN ^b^	SZT ^c^	SZS ^d^
1	5.84	3-Hexen-1-ol	807	0.01 ± 0.02	0.02 ± 0.01	0.02 ± 0.00	0.04 ± 0.01
2	8.01	Linalool	1083	0.32 ± 0.12	0.25 ± 0.12	0.17 ± 0.04	1.78 ± 0.32
3	8.78	Borneol	1162	0.00 ± 0.01	0.04 ± 0.02	-	-
4	8.96	4-Terpineol	1170	0.73 ± 0.09	0.34 ± 0.08	0.17 ± 0.00	0.07 ± 0.01
5	9.03	*p*-Cymene-8-ol	1175	0.24 ± 0.05	0.24 ± 0.03	0.03 ± 0.01	0.07 ± 0.03
	**Alcohols**		**Total**	**1.30**	**0.89**	**0.40**	**1.97**
6	4.17	Hexanal	734	0.04 ± 0.02	0.02 ± 0.01	0.01 ± 0.00	0.01 ± 0.00
7	6.70	Benzaldehyde	937	0.75 ± 0.04	1.43 ± 0.50	0.93 ± 0.03	0.49 ± 0.04
8	7.24	*n*-Octanal	984	0.42 ± 0.15	0.74 ± 0.16	1.98 ± 0.11	1.67 ± 0.29
9	8.09	Nonanal	1091	0.02 ± 0.00	0.05 ± 0.01	-	0.02 ± 0.00
10	9.59	Cumin aldehyde	1234	1.94 ± 0.36	1.04 ± 0.16	0.04 ± 0.03	0.54 ± 0.15
11	9.73	*p*-Anisaldehyde	1247	0.62 ± 0.15	0.23 ± 0.06	1.63 ± 0.11	7.51 ± 2.12
	**Aldehydes**		**Total**	**3.79**	**3.50**	**4.59**	**10.24**
12	6.48	Styrene	853	0.18 ± 0.14	0.36 ± 0.11	0.20 ± 0.12	0.46 ± 0.10
13	7.27	β-Cymene	1003	0.02 ± 0.01	0.02 ± 0.02	0.05 ± 0.01	0.06 ± 0.03
14	7.99	*p*-Dimethylstyrene	1080	0.42 ± 0.16	0.22 ± 0.01	0.11 ± 0.00	0.22 ± 0.05
15	11.81	α-Curcumene	1461	0.85 ± 0.36	0.56 ± 0.10	0.20 ± 0.05	0.13 ± 0.04
	**Aromatics**		**Total**	**1.46**	**1.17**	**0.56**	**0.88**
16	6.87	*n*-Caproic acid	955	0.72 ± 0.11	1.66 ± 0.09	0.43 ± 0.04	0.34 ± 0.03
17	8.74	*n*-Caprylic acid	1149	0.11 ± 0.04	0.21 ± 0.02	0.06 ± 0.01	0.04 ± 0.01
	**Carboxylic acids**		**Total**	**0.83**	**1.87**	**0.50**	**0.37**
18	9.49	Fenchyl acetate	1226	0.02 ± 0.01	0.02 ± 0.01	0.02 ± 0.02	0.01 ± 0.00
19	10.60	Terpinyl acetate	1339	0.24 ± 0.08	1.96 ± 0.25	0.01 ± 0.01	0.13 ± 0.03
20	10.84	Geranyl acetate	1364	0.19 ± 0.07	1.09 ± 0.00	0.01 ± 0.01	0.11 ± 0.02
21	11.36	Ethyl linoleate	1453	0.32 ± 0.06	0.16 ± 0.03	0.11 ± 0.01	0.03 ± 0.01
22	11.79	Methyl linolelaidate	1459	0.20 ± 0.09	0.06 ± 0.09	0.06 ± 0.01	0.03 ± 0.00
	**Esters**		**Total**	**0.97**	**3.30**	**0.21**	**0.31**
23	7.35	Cineole	1020	0.25 ± 0.09	0.60 ± 0.06	0.03 ± 0.02	0.21 ± 0.03
24	9.15	Estragole	1188	35.45 ± 1.17 ^c,d^	28.97 ± 4.26 ^c,d^	2.84 ± 0.97 ^a,b^	9.10 ± 2.45 ^a,b^
25	9.42	*O*-Methylthymol	1222	0.01 ± 0.00	0.51 ± 0.40	-	-
26	10.02	Anethole	1277	17.43 ± 1.99	7.51 ± 1.94	13.64 ± 0.10	25.40 ± 14.22
	**Ethers**		**Total**	**53.14**	**37.60**	**16.51**	**34.71**
27	4.69	Furfural	777	0.01 ± 0.02	0.13 ± 0.10	0.54 ± 0.07	0.52 ± 0.03
28	6.71	5-Methyl-2-Furaldehyde	942	1.62 ± 0.08	3.07 ± 0.42	1.67 ± 0.16	0.89 ± 0.04
29	7.64	Furaneol	1043	0.04 ± 0.01	0.07 ± 0.04	0.36 ± 0.08	0.12 ± 0.03
30	7.83	5-formylfurfural	1065	0.37 ± 0.00	2.11 ± 1.13	0.12 ± 0.02	0.13 ± 0.03
31	9.38	5-Hydroxymethylfurfural	1211	0.44 ± 0.18	4.59 ± 3.86	0.08 ± 0.04	0.12 ± 0.02
	**Furans**		**Total**	**2.48**	**9.96**	**2.76**	**1.78**
32	8.00	Fenchone	1082	0.03 ± 0.02	0.01 ± 0.01	0.01 ± 0.00	0.17 ± 0.01
33	8.19	Maltol	1103	0.38 ± 0.06	0.83 ± 0.42	0.03 ± 0.01	0.20 ± 0.01
34	8.64	Camphor	1141	0.47 ± 0.00	1.24 ± 0.17	0.02 ± 0.00	0.26 ± 0.04
35	9.16	Dihydrocarvone	1197	0.62 ± 0.06	0.48 ± 0.16	0.03 ± 0.02	0.19 ± 0.08
36	9.63	Carvone	1237	12.09 ± 3.30 ^c,d^	14.83 ± 5.58 ^c,d^	1.06 ± 0.30 ^a,b,d^	4.77 ± 1.15 ^a,b,c^
	**Ketones**		**Total**	**13.58**	**17.38**	**1.16**	**5.58**
37	6.52	α-Thujene	906	0.10 ± 0.00	0.43 ± 0.35	0.41 ± 0.37	0.99 ± 0.33
38	6.69	Camphene	925	0.04 ± 0.04	0.25 ± 0.35	0.07 ± 0.02	0.05 ± 0.04
39	6.93	β-Myrcene	961	6.95 ± 1.89 ^c,d^	7.62 ± 0.57 ^c,d^	19.49 ± 1.01 ^a,b^	16.15 ± 2.79 ^a,b^
40	7.23	β-Pinene	971	1.89 ± 0.47	2.08 ± 0.17	5.44 ± 0.27	4.54 ± 0.78
41	7.26	α-Terpinene	1002	0.21 ± 0.06	0.23 ± 0.02	0.60 ± 0.03	0.51 ± 0.08
42	7.31	D-Limonene	1016	6.44 ± 0.76 ^d^	6.06 ± 1.65 ^d^	5.55 ± 0.56 ^d^	9.06 ± 0.71 ^a,b,c^
43	7.35	β-cis-Ocimene	1027	0.18 ± 0.02	0.49 ± 0.00	0.04 ± 0.00	0.15 ± 0.02
44	7.45	β-Ocimene	1033	0.16 ± 0.04	0.37 ± 0.04	0.04 ± 0.00	0.17 ± 0.05
45	7.58	γ-Terpinene	1042	0.24 ± 0.11	0.27 ± 0.06	0.15 ± 0.02	0.06 ± 0.06
46	8.45	Neo-allo-ocimene	1117	0.03 ± 0.02	0.04 ± 0.02	0.01 ± 0.00	0.02 ± 0.00
	**Monoterpene hydrocarbons**		**Total**	**16.23**	**17.84**	**31.78**	**31.70**
47	4.68	2-Methylpyrazine	763	0.05 ± 0.08	0.09 ± 0.08	0.05 ± 0.03	0.03 ± 0.00
48	6.49	2,5-Dimethylpyrazine	876	0.06 ± 0.02	0.48 ± 0.23	0.47 ± 0.18	0.71 ± 0.46
49	7.70	α-Aminoxypropionic acid	1056	0.04 ± 0.06	0.07 ± 0.08	0.08 ± 0.00	0.03 ± 0.03
	**Nitrogen-containing compounds**		**Total**	**0.16**	**0.64**	**0.60**	**0.77**
50	7.82	Linalool oxide	1062	0.22 ± 0.01	1.26 ± 0.68	0.07 ± 0.01	0.10 ± 0.01
	**Oxides**		**Total**	**0.22**	**1.26**	**0.07**	**0.10**
51	10.10	Carvacrol	1287	3.91 ± 1.18 ^c^	2.67 ± 0.53 ^c^	37.43 ± 4.33 ^a,b,d^	8.84 ± 1.76 ^c^
	**Phenols**		**Total**	**3.91**	**2.67**	**37.43**	**8.84**
52	8.57	Pyranone	1133	0.02 ± 0.00	0.31 ± 0.25	0.01 ± 0.00	0.02 ± 0.01
	**Pyrans**		**Total**	**0.02**	**0.31**	**0.01**	**0.02**
53	7.25	Pyrrole-2-aldehyde	991	0.61 ± 0.16	0.63 ± 0.30	2.94 ± 0.13	2.49 ± 0.44
54	8.24	2-Formyl-1-methylpyrrole	1110	0.03 ± 0.02	0.18 ± 0.04	0.08 ± 0.01	0.10 ± 0.03
	**Pyrroles**		**Total**	**0.65**	**0.81**	**3.02**	**2.58**
55	10.53	δ-EIemene	1332	0.03 ± 0.01	0.11 ± 0.02	0.02 ± 0.00	0.02 ± 0.00
56	10.98	Bourbonene	1383	0.13 ± 0.05	0.18 ± 0.03	0.02 ± 0.01	0.04 ± 0.00
57	11.33	β-Caryophyllene	1416	1.03 ± 0.21	0.49 ± 0.11	0.36 ± 0.03	0.11 ± 0.02
58	11.34	Himachalene	1443	0.06 ± 0.02	0.02 ± 0.01	0.02 ± 0.00	-
	**Sesquiterpene hydrocarbons**		**Total**	**1.25**	**0.80**	**0.42**	**0.17**

Different letters (^a^, ^b^, ^c^, ^d^) indicate significant differences of the major characteristic constituents between groups.

**Table 2:** Phenolic compound contents (ug/g) of Egyptian dukkah and Syrian za’atar.

Compound	Rt (min)	EDT		EDN		SZT		SZS	
		Mean	SD	Mean	SD	Mean	SD	Mean	SD
Gallic acid	4.1	0.9 ^D^	0.00	2.0 ^C^	0.03	9.6 ^B^	0.03	21.3 ^A^	0.09
Protocatechuic acid	7	13.6 ^A^	0.22	5.2 ^B^	0.10	5.2 ^B^	0.00	2.8 ^C^	0.06
*p*-Hydroxybenzoic acid	10.3	11.3 ^A^	0.24	ND	ND	1.9 ^C^	0.04	5.6 ^B^	0.05
(Epi)Catechin	11.9	14.7 ^A^	0.16	ND	ND	14.8 ^A^	0.04	3.9 ^B^	0.53
Chlorogenic acid	12.8	2.37 ^B^	0.04	ND	ND	1.3 ^C^	0.00	2.5 ^A^	0.02
Caffeic acid	13.9	194.4 ^A^	0.19	31.0 ^D^	0.07	89.4 ^B^	0.28	63.6 ^C^	1.61
Syringic acid	14.9	23.1 ^A^	0.14	0.3 ^C^	0.00	1.5 ^B^	0.02	1.4 ^B^	0.09
Vanillic acid	16.7	2.8 ^B^	0.01	0.6 ^D^	0.02	3.9 ^A^	0.07	1.2 ^C^	0.05
Ferulic acid	21	325.8 ^A^	0.23	18.9 ^D^	0.07	91.3 ^B^	0.14	31.7 ^C^	0.26
Sinapic acid	21.8	28.8 ^A^	0.11	2.6 ^D^	0.05	5.4 ^B^	0.01	3.8 ^C^	0.11
Rutin	24.4	302.1 ^A^	0.89	12.5 ^D^	0.59	68.6 ^B^	0.43	29.0 ^C^	0.13
*p*-Coumaric acid	27.1	2.4 ^C^	0.06	4.1 ^B^	0.11	2.7 ^C^	0.09	10.5 ^A^	0.32
Apigenin-7-*O*-glucoside	28.8	7.8	0.29	ND	ND	ND	ND	ND	ND
Rosmarinic acid	30.2	338.3 ^A^	1.62	22.6 ^D^	0.38	149.6 ^B^	0.06	34.7 ^C^	0.33
Daidzein	34	26.8 ^A^	0.22	1.4 ^C^	0.11	ND	ND	5.1 ^B^	0.13
Cinnamic acid	35.6	11.0 ^A^	0.03	1.1 ^C^	0.02	1.3 ^B^	0.02	0.3 ^D^	0.03
Quercetin	36.4	3.9 ^C^	0.00	4.4 ^B^	0.04	5.6 ^A^	0.17	5.7 ^A^	0.13
Genistin	39	9.7 ^A^	0.04	3.2 ^C^	0.08	4.9 ^B^	0.01	ND	ND
Kaempferol	40.8	2.8 ^A^	0.07	ND	ND	1.1 ^B^	0.06	1.1 ^B^	0.00

Means with the same letter (superscript) in raw are not significantly different at (*p* > 0.05). ND: not detected.

**Table 3:** Vapor-phase antibacterial activity of the tested herbal mixtures and their major individuals.

Vapor-Phase Minimum Inhibitory Concentration (VP-MIC) mg/mL									
Mix	*Enterococcus faecalis* *ATCC19433*	*Staphylococcus aureus Newman*	*MRSA USA300*	*Klebsiella pneumoniae ATCC13883*	*Acinetobacter baumannii AB5075*	*Pseudomonas aeruginosa PAO1*	*Enterobacter cloacae*	*Escherichia coli ATCC87*	*Salmonella typhi ATCC* *35664*
Th	*	1.8 ± 1	2.4 ± 0	2 ± 0.7	*	1.2 ± 1	4.7 ± 0	2 ± 0.7	4.7 ± 0
Su	*	2.4 ± 0	2.4 ± 0	1.6 ± 0.7	4.7 ± 0	2.4 ± 2	*	3.1 ± 1.4	2 ± 0.7
Cr	*	1.2 ± 0	1.2 ± 0	1.2 ± 0	2.4 ± 2	1.2 ± 0	1.6 ± 0.7	2 ± 0.7	1 ± 0.3
Cp	*	3.5 ± 2	*	3.5 ± 2	*	*	*	4.7 ± 0	4.7 ± 0
EDT	*	2.4 ± 0	2.4 ± 0	2.4 ± 0	2.4 ± 0	2.4 ± 0	3.1 ± 1.4	4.7 ± 0	2.4 ± 0
EDN	3.1 ± 1.4	0.8 ± 0.3	0.8 ± 0.3	3.1 ± 1.4	2.4 ± 0	1.2 ± 0	2.0 ± 0.7	0.8 ± 0.3	0.8 ± 0.3
SZT	*	2.4 ± 0	2.4 ± 0	2.4 ± 0	2.4 ± 0	2.4 ± 0	*	2.4 ± 0	2.4 ± 0
SZS	*	4.7 ± 0	4.7 ± 0	4.7 ± 0	4.7 ± 0	4.7 ± 0	*	4.7 ± 0	4.7 ± 0

*—Indicates that there was no antibacterial activity detected within the tested concentrations.

**Table 4:** Antioxidant potential of the tested herbal mixtures.

Tested Mixtures	DPPH Assay	ABTS Assay	ORAC Assay	FRAP Assay	Ferrozine Iron Metal Chelation Assay
	μM T eq/mg	μM T eq/mg	μM T eq/mg	μM T eq/mg	μM EDTA eq/mg
EDT	59.93 ± 1.99	225.32 ± 6.15	1577.86 ± 50.14	145.26 ± 4.32	51.69 ± 5.33
EDN	26.92 ± 0.75	125.15 ± 5.92	1303.68 ± 92.02	59.89 ± 2.33	22.26 ± 2.38
SZS	86.79 ± 1.38	263.52 ± 9.7	1138.11 ± 83.03	144.81 ± 7.95	19.30 ± 0.52
SZT	104.10 ± 1.68	337.26 ± 13.84	1479.09 ± 88.19	212.75 ± 12.85	18.08 ± 1.62

## Data Availability

Not applicable.

## Sample Availability

Samples of the compounds are available from the authors.
